# An innovative alternative for spherophakia

**DOI:** 10.3205/oc000180

**Published:** 2021-03-29

**Authors:** Madhivanan Nivean, Devi Pratheeba Nivean, Rithula Raja

**Affiliations:** 1 M. N. Eye Hospital, Chennai, India

**Keywords:** spherophakia, aphakic IoL, CM T-flex IOL, glued IOL

## Abstract

**Objective:** The aim of this case report is to report a new aphakic intraocular lens (IOL) that can be used for spherophakia.

**Methods:** This is a single case report wherein the authors elaborate the technique of inserting the new IOL design in patients with spherophakia.

**Results:** This new IOL design is very stable and is very promising in our follow-up of 6 months.

**Conclusion:** The CM T-flex IOL can be a simple and alternate option for correcting aphakia.

## Introduction

Spherophakia is a rare diagnosis which is often associated with a shallow anterior chamber, angle-closure glaucoma, lens subluxation and lenticular myopia [1]. In this condition, the crystalline lens has a reduced equatorial diameter and an expanded anterior-posterior diameter [2]. When cataracts occur with subluxation of the lens, vision is often markedly affected. This becomes challenging to the surgeon to give good visual outcomes and to minimize potential complications [3]. We present this case as we had used a new aphakic IOL designed by the authors.

## Case description

A 23-year-old female presented with painless progressive decrease in vision in both eyes over the past 2 years. The defective vision was not associated with trauma or any other systemic illness.

**Figure 1:**
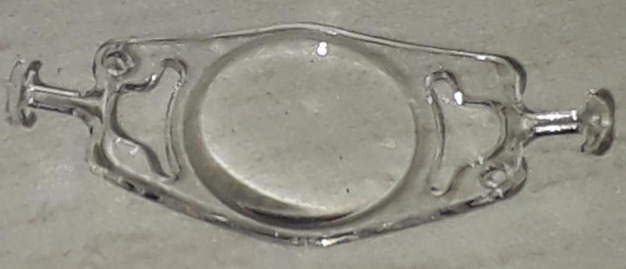
External photograph of the CM T-flex IOL

On examination, the best corrected visual acuity (BCVA) was 2/60 in both eyes. Both eyes had subluxated cataractous lens (nuclear sclerosis grade II, Lens Opacities Classification System (LOCS) III classification) with nine clock hours of subluxation (3–12 o’clock) causing a shallow anterior chamber. The lenses in both eyes were spherical, had reduced equatorial diameter and increased anteroposterior diameter suggestive of spherophakia. The pupil was circular with normal iris pattern and pupillary reactions. The intraocular pressure (IOP) was 16 mm Hg in the right eye and 14 mm Hg in the left eye. Posterior segment was normal in both eyes. A clinical diagnosis of bilateral spherophakia with cataract was made and lensectomy with vitrectomy and CM T-flex aphakic IOL placement (Figure 1 (Fig. 1)) under local anaesthesia was planned. After all the necessary routine pre-operative evaluations, the patient underwent the procedure in the left eye first.

Under peribulbar anaesthesia, using the Ashwin Glued IOL marker 0 -1800 is marked. This is an important step as it ensures the centration and torsional stability of the IOL [4], [5]. Conjunctival peritomy is done on either side and bipolar cautery is used to cauterize the bleeders. Two partial thickness limbal based scleral flaps of about 2.5 mm x 2.5 mm are created on either side of the markings. A 23-gauge trocar is placed in the inferotemporal quadrant for the infusion to prevent hypotony during the procedure. Using the 23-gauge cutter, lensectomy is performed through the side port, diluted triamcinolone acetonide is injected, and anterior vitrectomy is completed. Two sclerotomies are made with a 23-gauge needle, 1.5 mm from the limbus on either side under the sclera flap. A 2.8 mm clear corneal incision is made using the keratome. The CM T-flex aphakic IOL is a foldable hydrophilic lens with a specialized T-shaped haptics which is loaded in the cartridge and placed in the injector.

**Figure 2:**
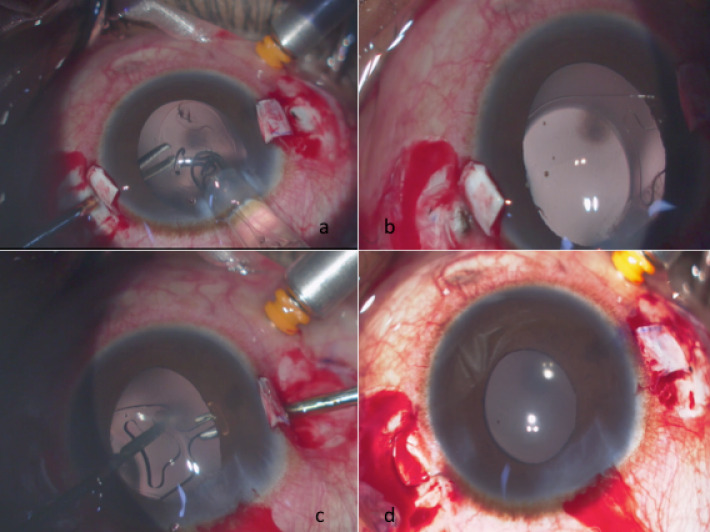
Showing grasping of the T-junction with the PraNIv T-flex forceps (A), exteriorized leading haptic under the scleral flap (B), grasping of the lagging haptic by the handshake technique (C) and the exteriorized lagging haptic (D)

The IOL is injected through the cornea gently so that the T-junction of the IOL comes out first, which makes it easy to grasp using the specially designed PraNiv T-flex forceps through one sclerostomy. The forceps has a specially designed short and broad tooth to hold the IOL without causing any damage or chipping. The leading haptic of the IOL is gently brought out through the sclerostomy. The lagging haptic is then positioned on the iris tissue. The arm of the haptic over the iris is then grasped by the Nishi grasping forceps (which has cris-cross serrations for a firm grip), and the trailing T-junction is transferred to the other hand by the PraNiv T-flex forceps using a hand shake technique, and exteriorized through the other sclerostomy. We can see the pop of the T-haptic after pulling it out (Figure 2a, b, c, d (Fig. 2)). The specially designed T-shaped haptic of the IOL prevents it from slipping back into the eye. The anterior chamber is formed well and the infusion cannula is removed, the sclera bed is made dry, and fibrin glue is used to seal the sclera flap and the conjunctiva. Antibiotic steroid eye drops are prescribed in tapering doses postoperatively. At one month post–op, the patient’s vision improved to 6/9, N8 in the left eye. The anterior chamber was well-formed with no reaction, and the IOL was found to be well-centred in the pupillary axis. The surgery for the right eye was planned and carried out in the same manner. At the final review at one month, the patient had a best corrected vision of 6/9, N 8 with a well-centred IOL. The patient has been on regular follow-up with us for the past 6 months. She is doing extremely well and maintaining an unaided vision of 6/9 in both eyes.

## Discussion

Spherophakia is a rare congenital bilateral eye disorder, which presents with weak zonules around a smaller and more spherical crystalline lens with an increased anteroposterior thickness of the lens and highly myopic eye [2]. The lens zonules are developmentally hypoplastic and abnormally weak.

Due to nonattachment of the posterior zonules to the equatorial zone, the normal lens becomes spherical. The lens may undergo subluxation or dislocation from the patellar fossa, leading to defective accommodation. The disease can present as an isolated condition or may run in families, and such cases have been reported in multiple lineage studies [6]. Subluxation of the lens may occur anteriorly, inferiorly or posteriorly [7] and may lead to pupillary block glaucoma [8].

Lensectomy has been previously described as an option for managing the dislocated lens [9]. The choice of the IOL depends largely on the surgeon and on patient factors. Angle-supported anterior chamber lenses (ACIOL) and iris-enclavated lenses [10] are commonly used. Posterior chamber IOL (PCIOL) with/without capsule tension rings (CTR) [11] and scleral-fixated IOL (SFIOL) [12] have also been described in various case reports. However, the usage of CTR depends on the degree of subluxation.

Angle-supported ACIOLs have been reported to be associated with corneal endothelial cell loss, peripheral anterior synechiae (PAS) formation and glaucoma due to chronic anterior chamber irritation [13].

Iris-enclavated lenses may be placed anterior or posterior to the iris. But these iris claw lenses are hinging on a light sensitive mobile structure [14].

PCIOL placement is controversial as the zonules are developmentally weak and there is a possibility of the bag lens complex falling into the vitreous. Khokhar et al. [15] have described a ‘dual-support technique’ of insertion of CTS (capsule tension strip) with CTR in a case of spherophakia and have opined that this may help overcome zonular weakness.

SFIOL has been documented as a viable option, however it requires an effective vitrectomy and proper scleral tunnel fixation. SFIOL haptics have to be buried under the scleral flaps with polypropylene sutures, and reports have shown that due to the lack of fibrosis around the lens loops, the suture is the only support for the lens [16]. Transscleral suture exposure has been reported at 14.7–17.9%. There is a possibility of late decentration caused by suture degradation even years after, thus SFIOL proves to be a difficult procedure technically and may be associated with reported complications [17], [18].

The special T-shaped haptics of the new CM T-flex aphakic IOL lens design help with stability and prevent the lens from falling back inside. There is no need for suture or tucking of the haptics, which helps in reducing the surgical time. The new and innovative lens design ensures good centration as the lens is fixed in one place and there is no risk of torsion. Due to the angulation between the optic and the haptics, friction between the IOL and the iris is prevented. This reduces iritis and ensures a good dilated pupil for post-op fundus evaluation.

## Conclusion

The CM T-flex aphakic IOL is a new aphakic IOL design. In the case series of our patients we found it to be a safe and highly effective alternative which simplifies management and ensures optimal outcomes with a shorter learning curve, shorter intraoperative time and minimal complication.

## Notes

### Manufacturer details

Appasamy Associates, Chennai, India

### Specifications of IOL

- Material: hydrophilic 26% water
- Refractive index: 1.460
- Optic diameter: 6.00 mm
- Overall diameter: 13.75 mm
- Angulation: 10 degree
- A constant: 118.0

### Competing interests

The authors declare that they have no competing interests.
